# Rapidly Disseminated Kaposi’s Sarcoma Despite Initiation of Antiretroviral Therapy

**DOI:** 10.7759/cureus.39627

**Published:** 2023-05-29

**Authors:** Chantal Saberian, Breanna R Campbell

**Affiliations:** 1 Department of Medicine, Baton Rouge General, Baton Rouge, USA; 2 Department of Infectious Diseases, Baton Rouge General, Baton Rouge, USA

**Keywords:** aids, hiv, antiretroviral therapy, kaposi's sarcoma, visceral kaposi sarcoma, kshv inflammatory cytokine syndrome (kics), combination antiretroviral therapy, hiv aids, kaposi's sarcoma-associated herpesvirus (kshv)

## Abstract

Kaposi’s sarcoma (KS) is the most common malignancy in people living with HIV. The reported incidence of AIDS-related KS has been dramatically decreased with the introduction of antiretroviral therapy (ART). Systemic treatment with ART is indicated for patients with AIDS-related KS; however, some patients may develop KS-related immune reconstitution inflammatory syndrome characterized by sudden rapid progression of new or pre-existing KS within the initiation of ART. Here, we present a case of rapidly disseminated KS with widespread visceral involvement despite ART initiation in a 27-year-old African American man with advanced HIV/AIDS.

## Introduction

Kaposi’s sarcoma (KS) is a low-grade vascular tumor caused by human herpesvirus 8 (HHV-8) [1]. It is the most frequent malignancy in people living with human immunodeficiency virus/acquired immunodeficiency syndrome (HIV/AIDS) [2]. AIDS-related KS has an unpredictable clinical course, ranging from minimal disease to a rapidly progressing malignancy associated with high mortality depending on the sites of involvement [2]. KS primarily affects the skin of the face, trunk, and upper and lower extremities [3]. Mucosal involvement (oral cavity, oropharyngeal, and genital mucosa) is identified in 20% of patients; other organ spread, such as the lung, gastrointestinal tract, and lymph node, is noted in the disseminated form of KS [4]. The incidence of AIDS-related KS has been dramatically decreased with the introduction of antiretroviral therapy (ART), the cornerstone AIDS-related KS treatment [5]. The regression or resolution of the KS lesions occurs within several months in 20% to 80% of patients after the ART commencement paralleling the increase in the CD4 counts and decreasing the viral load [6-8]. However, some patients may experience KS-related immune reconstitution inflammatory syndrome (IRIS)-like syndrome that could exacerbate the pre-existing lesions after the initiation of ART [9].

## Case presentation

Mr. DD is a 27-year-old African American man who was diagnosed with AIDS and cryptococcal meningitis at the age of 14 years. His clinical course was complicated by difficulties with adherence and engagement in care due to his mental state, polysubstance use disorder, and poor social support. He was diagnosed with biopsy-proven cutaneous KS in late 2021, two months prior to admission to our institution. At that time, Mr. DD’s CD4 count was 12 cells/μL and his HIV viral load was 100,000 copies/mL. He was initiated on bictegravir/emtricitabine/tenofovir alafenamide as well as monthly inhaled pentamidine. He was previously on suppressive trimethoprim/sulfamethoxazole, but this was discontinued in the setting of thrombocytopenia. He was previously on outpatient suppressive fluconazole for his prior history of cryptococcal meningitis, recently held due to transaminitis. Finally, Mr. DD was on valganciclovir for cytomegalovirus (CMV)-induced esophagitis.

**Figure 1:**
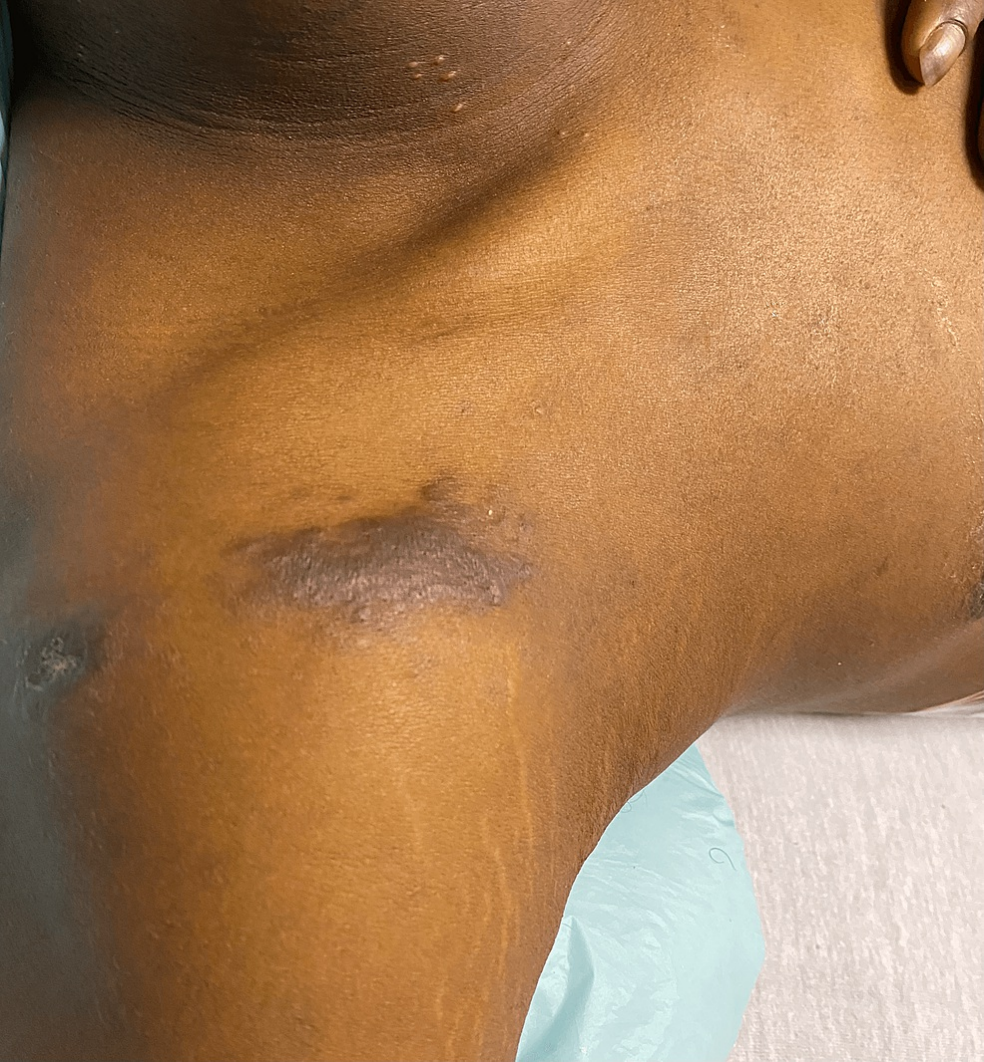
Initial presentation with Kaposi’s sarcoma on the anterior shoulder

He was admitted to our institution with a three-week history of sharp, constant, and severe abdominal pain with radiation to the back, associated with nausea/vomiting and poor oral intake. His vital signs on arrival included a blood pressure of 133/90 mmHg, heart rate of 100 beats per minute, a body temperature of 98.5°F, respiratory rate of 18 breaths per minute, and oxygen saturation (SpO2) of 98% on room air. Physical examination was remarkable for icteric sclera, moderate right upper quadrant abdominal pain, and lesions consistent with KS on the right anterior shoulder (Figure 1). No oral lesions were noted, and the bilateral lungs were clear on auscultation.

A review of his outpatient records disclosed that his CD4 count and HIV viral load had improved to 116 cells/μL and 70,000 copies/mL, respectively, one month prior to admission, and his HIV viral load further improved to 40,000 copies/mL at the time of admission. His CD4 count at admission was unchanged. His white blood cell count was 4.62 K/μL, hemoglobin was 9.2 g/dL, and platelets count was 208 K/μL. Additional labs included total bilirubin at 9.2 mg/dL, direct bilirubin at 7.2 mg/dL, alkaline phosphatase at 1132 U/L, aspartate aminotransferase at 394 U/L, alanine aminotransferase at 250 U/L, albumin at 2.4 g/dL, lipase at 17 U/L, and international normalized ratio of 1.2. Infectious workup, including tuberculin skin test, serum cryptococcal antigen, urine histoplasma antigen, stool *Cryptosporidium*, stool *Isospora*, stool Giardia, hepatitis A, B, and C, CMV, and Epstein-Barr virus serology, were negative. A right upper quadrant ultrasound showed gallbladder thickening with pericholecystic fluid. Subsequent magnetic resonance cholangiopancreatography (MRCP) was pursued for his cholestatic picture. The MRCP showed cholelithiasis and biliary sludge but no biliary ductal dilatation; there was no excretion into the normal caliber bile ducts on the MRCP (consistent with abnormal hepatic retention). Hepatobiliary iminodiacetic acid showed hepatic dysfunction with retention of the radiopharmaceutical. A percutaneous cholecystostomy tube was placed. Nonetheless, hyperbilirubinemia and alkaline phosphatemia continued to rise. He underwent endoscopic retrograde cholangiopancreatography and sphincterotomy for possible AIDS cholangiopathy.

A month into his hospital course, Mr. DD then developed a new onset nonproductive cough, wheezing, and dyspnea. CT of the chest showed consolidation of the lower lung fields concerning for pulmonary KS. Sputum sampling for *Pneumocystis jirovecii *pneumonia (PJP) was negative. Our pulmonary service performed a bronchoscopy with gross findings of diffuse, impressive, and patchy mucosal abnormalities with cobblestoning and hyperemia, consistent with KS. Given the potential for bleeding, biopsies were not taken. Bronchoalveolar lavage showed pale yellow fluid with white blood cells of 197 cells/uL (differential cell count of neutrophils 1%, lymphocytes 89%, monocytes 10%, and eosinophils 0%), while polymerase chain reaction, bacterial, fungal, and acid-fast testing were also negative.

**Figure 2:**
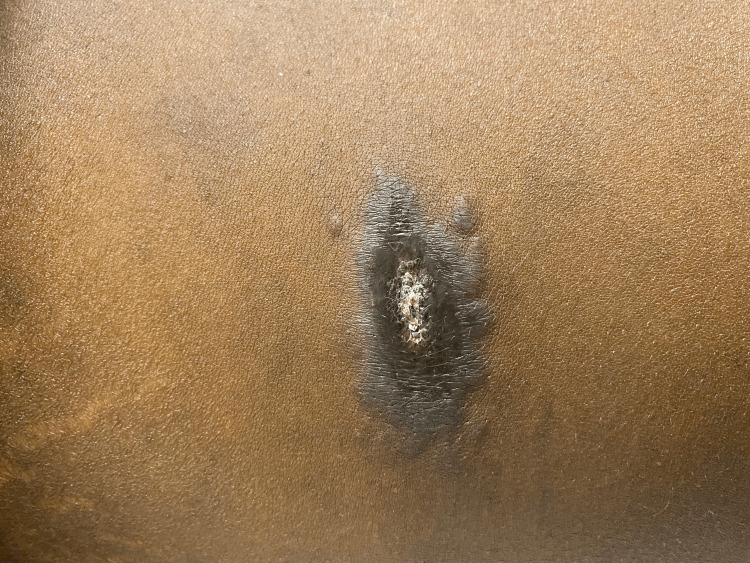
A violaceous plaque on the abdomen

**Figure 3:**
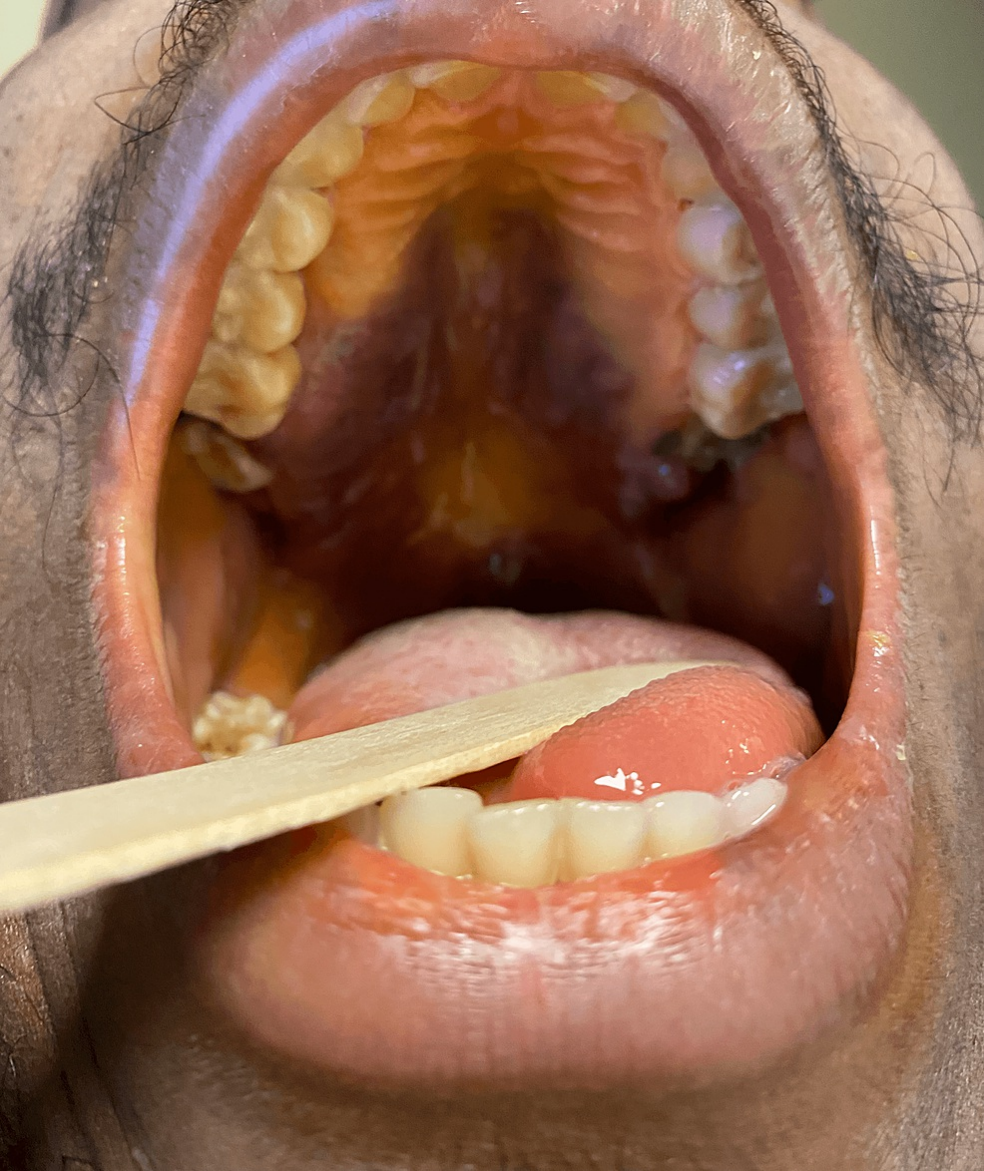
Violaceous lesions on the hard palate

Throughout his hospitalization, the patient was maintained on his ART regimen. He developed two new violaceous patches over the abdomen while inpatient (approximately a month into his hospital course), presumably more KS (Figure 2) (declined biopsy), and new hard palate violaceous lesions consistent with KS (Figure 3) (declined biopsy). Oncology was consulted and Mr. DD underwent echocardiography and CT of the chest in anticipation of doxorubicin therapy. Liver and bone marrow biopsies to confirm KS were offered to the patient, which he declined. Mediport placement was planned for chemotherapy initiation but postponed in the setting of fever. Subsequent blood cultures identified *Enterococcus faecalis*. He initially received empiric vancomycin plus piperacillin-tazobactam, which later was tailored to ampicillin based on culture results. After repeat blood cultures were cleared, a mediport was placed for doxorubicin administration. Unfortunately, Mr. DD developed recurrent fever post-chemotherapy, and blood cultures showed *Escherichia coli*. Piperacillin-tazobactam was started. CT of the abdomen/pelvis did not show an infectious process, and the cholecystostomy tube was removed.

Almost two months into his hospital course, Mr. DD was admitted to the intensive care unit with fever, acute hypoxic respiratory failure, and encephalopathy. Chest X-ray showed ongoing lung involvement with loculated right pleural effusion. Mr. DD was intubated and underwent bronchoscopy, which revealed heavy blood and mucous secretions; after 10-15 cc lavage, he rapidly desaturated to SpO2 of 70%, and the procedure was aborted. A repeat PJP smear was negative. Despite maximal settings on ventilation, the patient had persistent hypoxia. Consent was obtained and thoracentesis was done, which removed hemorrhagic pleural fluid, the cytology of which was negative for malignancy. All infectious studies (bacterial, fungal, and acid-fast testing) were negative. Two days later, the patient was found in asystole, and he was pronounced dead.

## Discussion

Overall, the clinical picture of our patient was consistent with rapidly disseminated KS despite the initiation of ART, concerning for KS-IRIS. Diagnosis of disseminated KS can be challenging due to overlapping symptoms and findings with other opportunistic infections in advanced HIV/AIDS patients. His initial presentation with abdominal pain and cholestatic transaminitis was suspicious for AIDS cholangiopathy, which can be caused by several different opportunistic infections, including KS-associated herpesvirus, CMV, as well as *Cryptosporidium parvum*, *Enterocytozoon bieneusi*, *Isospora*, *Cyclospora*, *Mycobacterium avium complex*, *Cryptococcus*, and PJP [10]. Targeting these etiologies does not usually improve AIDS cholangiopathy, which is often treated with relief of obstruction where possible and continued ART [10,11]. While we did not confirm AIDS cholangiopathy, he underwent sphincterotomy during his procedure to offer empiric treatment.

The cornerstone for the treatment of AIDS-related KS is ART, but the response to ART differs greatly [12]. The regression or resolution of the KS lesions occurs within several months in 20% to 80% of patients after the ART commencement paralleling the increase in the CD4 counts and decreasing the viral load [6-8]. Our patient rapidly deteriorated within approximately two months of ART initiation. His suppressed viremia after initiating therapy indicates that he was indeed adherent (at least recently) to his medication. We, therefore, presumed KS-IRIS was possible in this patient, though his CD4 count was not impressively improved. It is possible the CD4 was suppressed in the setting of valganciclovir-induced neutropenia [13]. A randomized controlled trial showed that approximately 60% of patients with pre-existing KS developed IRIS after starting ART [14], which can then cause rapid progression of the lesions. This is just like our patient, who developed new violaceous patches over the skin and hard palate accompanied by worsening respiratory distress during hospitalization, which correlates with the disseminating nature of the disease. Lung involvement occurs in approximately 45% of patients with AIDS-related KS. It is a life-threatening form of KS [15]. Sometimes it is difficult to differentiate from other opportunistic infections such as *Mycobacterium avium complex*, *Cryptococcus*, or PJP with pulmonary involvement. Bronchoscopy was performed on this patient, which showed both blood and pus. Perhaps our patient had diffuse alveolar hemorrhage secondary to KS or any other infection; pus would raise suspicion for the additional bacterial process. He was also at high risk for PJP given the inability to properly prophylax in the setting of hepatic dysfunction; also, he was at risk for typical and atypical community or hospital-acquired pneumonia as well as fungal infections.

Systemic steroid therapy was considered for the possibility of IRIS but held as steroids are associated with increased KS mortality [16,17]. Patients who do not respond to ART or who have extensive disease are treated with systemic therapy, mainly with pegylated liposomal doxorubicin, paclitaxel, and pomalidomide [18]. Our patient received one cycle of pegylated liposomal doxorubicin, but despite the treatment, the patient deteriorated very fast due to the aggressive and advanced nature of his disease.

## Conclusions

Although the development of improved ART has done much to reduce the incidence and improve the overall survival in patients with AIDS-related KS, our patient’s AIDS-related KS rapidly disseminated, with widespread visceral involvement despite ART and systemic chemotherapy initiation. There is an unmet need for new and effective treatments targeting AIDS-related KS.
